# Disease Control and Treatment Satisfaction in Patients with Chronic Spontaneous Urticaria in Japan

**DOI:** 10.3390/jcm13102967

**Published:** 2024-05-17

**Authors:** Atsushi Fukunaga, Yuko Kishi, Kazuhiko Arima, Hiroyuki Fujita

**Affiliations:** 1 Department of Dermatology, Division of Medicine for Function and Morphology of Sensory Organs, Faculty of Medicine, Osaka Medical and Pharmaceutical University, Osaka 569-8686, Japan; atsushi.fukunaga@ompu.ac.jp; 2 Specialty Care Medical, Sanofi K.K., Tokyo Opera City Tower, 3-20-2, Nishi Shinjuku, Tokyo 163-1488, Japan; kazuhiko.arima@sanofi.com (K.A.); hiroyuki2.fujita@sanofi.com (H.F.)

**Keywords:** chronic spontaneous urticaria, chronic urticaria, Japan, patient reported outcome measures, patient satisfaction

## Abstract

**Background:** Chronic spontaneous urticaria (CSU), characterized by the recurrence of pruritic hives and/or angioedema for >6 weeks with no identifiable trigger, has a negative impact on health-related quality of life (HRQoL). **Methods:** The objective of this web-based cross-sectional study was to evaluate disease control, disease burden, and treatment satisfaction in Japanese adults with CSU using the Urticaria Control Test (UCT), HRQoL outcomes, and the Treatment Satisfaction Questionnaire for Medication–9 items (TSQM-9). **Results:** In total, 529 adults were included in the analysis (59.9% female), with a mean ± standard deviation (SD) in CSU duration of 13.2 ± 13.0 years. Based on UCT scores, two-thirds of patients had poor (score of 0–7; 23.6%) or insufficient (score of 8–11; 43.3%) symptom control, and one-third had good control (score of 12–16; 33.1%). Overall treatment satisfaction was not high, with mean ± SD TSQM-9 scores of 55.5 ± 17.6% for effectiveness, 68.2 ± 18.8% for convenience, and 59.2 ± 18.4% for global satisfaction. No apparent differences in TSQM-9 scores were observed between patients receiving different medications. HRQoL outcomes were worse among patients with poor/insufficient symptom control. **Conclusions:** Japanese adults with CSU have a high disease burden, and better treatment options are needed to increase treatment satisfaction.

## 1. Introduction

Chronic spontaneous urticaria (CSU) is a chronic inflammatory skin disease characterized by the recurrence of wheals (hives), angioedema, or both for >6 weeks with no identifiable trigger [1,2]. According to epidemiological studies in Chinese and Korean populations, the point prevalence of urticaria ranges from 0.8% to 4.5% [3,4]. A Japanese epidemiological survey by Saito and colleagues reported that approximately two-thirds (66.8%) of patients with urticaria had CSU in 2020 [5]. The pathogenesis of CSU is not fully elucidated, and its duration can range from months to years [1].

In 2018, a Japanese real-world study by Itakura and colleagues showed that chronic urticaria (CU; including CSU) was associated with an impaired health-related quality of life (HRQoL) and reduced work productivity, similar to that experienced with psoriasis or atopic dermatitis [6]. Many patients with CU also reported low satisfaction regarding their condition and treatment [6]. Such findings highlight the unmet needs of affected patients, including the need to improve treatment options in this setting.

According to the 2018 Japanese guidelines for the management of urticaria, first-line treatment options include oral, non-sedating, or second-generation antihistamines (H1 receptor antagonists), while second-line options include the addition of H2 receptor or leukotriene receptor antagonists, tranexamic acid, diaphenylsulfone, anxiolytics, glycyrrhizin, neutropin (i.e., vaccinia virus-inoculated rabbit inflamed skin extract), or Chinese herbal medicine for those with persistent symptoms [1]. The international joint-initiative guidelines from the European Academy of Allergology and Clinical Immunology (EAACI), Global Allergy and Asthma European Network (GA^2^LEN), European Dermatology Forum (EuroGuiDerm), and Asia Pacific Association of Allergy, Asthma, and Clinical Immunology (APAAACI) similarly recommend the use of standard-dosed modern second-generation H1 receptor antagonists in patients with CU, but do not recommend the combined use of H1 and H2 receptor antagonists [2]. For patients who do not respond to antihistamines or the abovementioned second-line treatments, third-line options include omalizumab, cyclosporine, and short-term low-dose oral corticosteroids [1].

The anti-immunoglobulin E monoclonal antibody omalizumab was approved for the management of refractory CSU in Japan in March 2017 [7], and its effectiveness and safety have been confirmed in routine clinical practice [8]. In a previous study of 90 Japanese adults with CSU by Kaneko and colleagues, global treatment satisfaction with omalizumab was 77.6% (compared with 72.2% with antihistamines) [9]. However, this study reported lower patient-perceived convenience with omalizumab versus antihistamines [9], most likely due to the administration route (subcutaneous injection) of the biologic agent.

Although previous studies have evaluated the disease burden and treatment satisfaction among adults with CSU, they included a small number of enrolled patients and were conducted in a controlled clinical setting (i.e., specialist dermatology departments) [9]. Therefore, there is a need to examine more broadly the relationship between CSU disease control, disease burden, and treatment satisfaction in Japan, particularly as new treatments have emerged, such as biologics, and updated international and Japanese guidelines on urticaria management have been developed and disseminated [1,2]. Therefore, we conducted an online questionnaire to evaluate current CSU disease control, disease burden, and treatment satisfaction among Japanese adults with CSU.

## 2. Materials and Methods

### 2.1. Study Design

This web-based, cross-sectional, observational study of patients with CSU was conducted in Japan from 4–25 April 2022 (UMIN-Clinical Trials Registry: UMIN000047616). Individuals who were registered on the 2021 general consumer panel of Rakuten Insight Inc. (a market research company) were invited to voluntarily participate in the survey. An email with a link to the online questionnaire, which was open for 1 month between April and May 2022, was sent to members of the consumer panel. All responses received within the survey period were accepted, collected as primary data, and anonymized by Rakuten Insight, Inc.

The study was conducted in accordance with the principles of the Declaration of Helsinki and the Ethical Guidelines for Medical and Health Research Involving Human Subjects in Japan, issued by the Ministry of Health, Labour, and Welfare, the Ministry of Education, Culture, Sports, Science, and Technology, and the Ministry of Economy, Trade, and Industry.

### 2.2. Study Participants

Study participants were defined based on their survey responses. Adults (aged ≥ 20 years) with CSU who lived in Japan were eligible to participate. Participants who responded that they had previously been diagnosed with CSU or CU by a physician in the past, had urticaria symptoms for >6 weeks with no previous trigger, and had received treatment for CSU or CU in the 3 months prior to completing this survey were included. Because the diagnosis of CSU is not widespread in Japan, the diagnosis of CSU or CU was included in the selection criteria, and data from patients with CU were collected only if they had experienced symptoms for >6 weeks with no previous trigger. Patients may have also been diagnosed with allergic urticaria (i.e., an allergen/immunoglobulin E [IgE]-mediated urticaria subtype that occurs due to exposure to food, drugs, plants (including natural rubber products), insect toxins, etc., as defined by the Japanese guidelines [1]), acute urticaria, or cold urticaria. Patients who had only received over-the-counter medications and had not recently visited a hospital were also included.

Participants provided their informed consent before completing the questionnaire. Those who provided invalid responses were excluded from the analysis.

### 2.3. Study Objectives

The study objective was to describe treatment satisfaction among adults with CSU using the Treatment Satisfaction Questionnaire for Medication–9 items (TSQM-9). TSQM-9 scores were evaluated according to disease control (based on Urticaria Control Test [UCT] scores), CSU symptoms, and current/previous medications. Another objective was to describe the disease burden in adults with CSU using the following patient-reported outcomes (PROs): UCT, Numerical Rating Scale (NRS) for pruritus, burning, and sleep disturbance, Dermatology Life Quality Index (DLQI), Short Form-8 item (SF-8) health survey, and Work Productivity and Activity Impairment (WPAI) scores.

### 2.4. Online Questionnaire

The questionnaire included items regarding the participants’ demographics, disease characteristics, treatments, and the following six PROs: (1) TSQM-9 (range 0–100%) [10], which is a validated tool that evaluates treatment satisfaction using nine questions across the effectiveness, convenience, and global satisfaction domains (higher scores indicated higher satisfaction for that domain) (of note, the TSQM-9 omits the treatment-related adverse effects domain of the TSQM due to its potential to influence outcomes in real-world studies); (2) UCT (range 0–16 points) [11], which is a validated PRO that includes four questions to comprehensively evaluate control of urticaria symptoms over a 4-week period (scores of 0–7 = poor symptom control; 8–11 = insufficient symptom control; 12–16 = good symptom control) [6]; (3) NRS for pruritus, burning, and sleep disturbance (range 0–10 points), which rates the average and peak intensity (severity) of symptoms over the last 7 days (scores of 0 = none; 1–3 = mild; 4–6 = moderate; 7–9 = severe; 10 = very severe); (4) DLQI (range 0–30 points) [12], which is a 10-item questionnaire designed to assess the impact of the disease on HRQoL (scores of 0–1 = no effect; 2–5 = small effect; 6–10 = moderate effect; 11–20 = large effect; 21–30 = extremely large effect); (5) SF-8 (range 0–100 points) [13], which is an abbreviated version of the original SF-36 health survey, and measures HRQoL over the past 1 month in two standardized domains (physical health summary and mental health summary; higher scores indicate improved HRQoL (of note, the Japanese national standard mean SF-8 score is 50)); and (6) WPAI questionnaire (range 0–100%) [14], which is a validated instrument used to assess the impact of disease on work and productivity impairment over the last 7 days with regard to absenteeism (working hours lost due to CSU), presenteeism (impaired work due to CSU), overall work productivity loss, and daily activity impairment.

### 2.5. Statistical Analysis

Based on the total number of individuals in the Rakuten Insight, Inc. general consumer panel for 2021 (approximately 2,200,000), we empirically estimated that 250,000 responses to the screening questions would be collected. We assumed that ≥350 responses would meet the inclusion criteria based on a small-scale feasibility assessment. Considering the limitations of the study’s cost including the scale of licenses required, the target number for analysis was 500; a maximum of 550 responses were collected.

Study outcomes were assessed using summary statistics (mean, standard deviation [SD], median, interquartile range, and minimum/maximum values). Descriptive statistics were supplemented by additional testing to assist with data interpretation by identifying potentially clinically significant differences. The Mann–Whitney U test was used for pairwise comparisons, the Kruskal–Wallis test was used to test for significant differences between UCT subgroups, the Jonckheere–Terpstra test was used to identify increasing or decreasing trends across the three UCT subgroups, and the Steel–Dwass test was used for multiple comparisons between subgroups; all tests were conducted with a significance level of 5%.

Data processing and statistical analysis were conducted using Microsoft Excel 2013/2016, BellCurve Hideyoshi Dplus, version 1.12 (Social Information Service Co., Ltd., Tokyo, Japan), and Excel Statistics, version 3.23 (Social Information Service Co., Ltd., Tokyo, Japan).

## 3. Results

### 3.1. Study Population

**Figure 1:**
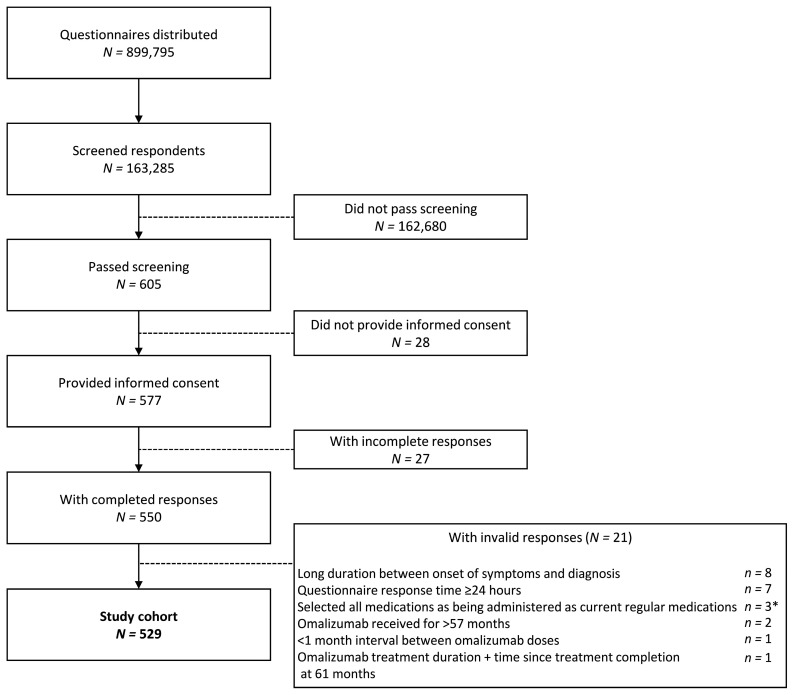
Disposition of study population. * Includes one patient with a response time of ≥24 h.

Online questionnaire responses were collected from 163,285 individuals in the general population, of whom 605 adults met the study definition of CSU (corresponding to a point-prevalence of CSU of 0.4%). In total, 550 adults with CSU met the inclusion criteria, provided informed consent, and gave complete responses (Figure 1). After excluding 21 individuals due to invalid responses, 529 participants with CSU were included in the final analysis.

Patients had a mean ± SD age of 45.3 ± 13.2 years, and 59.9% of the study population were female (Table 1). The mean ± SD duration of CSU was 13.2 ± 13.0 years, and 38.9% of the population (*n* = 206) had a history of CSU of ≥10 years. Only 16.4% reported a diagnosis of CSU, while most patients (93.8%) reported a diagnosis of CU. Of these patients with CU or CSU, 42.5%, 31.0%, and 18.0% also reported a diagnosis of allergic urticaria, acute urticaria, and cold urticaria, respectively. A previous or current history of angioedema was reported in 64 patients (12.1%). Other common comorbidities for which patients were currently receiving treatment included allergic rhinitis (*n* = 143; 27.0%), atopic dermatitis (*n* = 95; 18.0%), and hypertension (*n* = 84; 15.9%; Supplementary Table S1).

### 3.2. Disease Control

**Figure 2:**
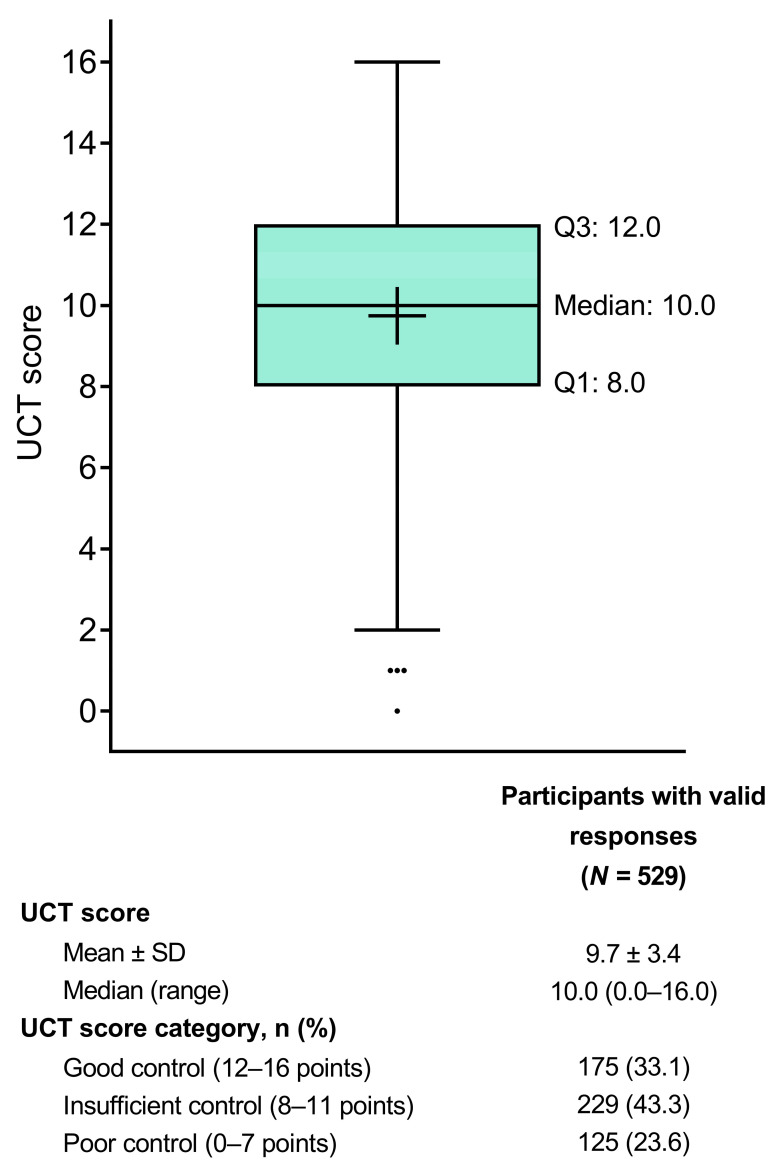
Tukey box plot of UCT scores indicating the status of urticaria and angioedema over the past 4 weeks. The box represents the lower quartile (Q1), median, and upper quartile (Q3) values, the cross represents the mean value, the error bars represent the minimum/maximum values (excluding outliers), and the dots represent the outliers. SD = standard deviation; UCT = Urticaria Control Test.

Patients had a mean ± SD UCT score of 9.7 ± 3.4 (Table 1; Figure 2). Based on their UCT scores, 175 patients (33.1%) had good symptom control (score of 12–16) over the 4 weeks prior to study participation, 229 (43.3%) had insufficient symptom control (score of 8–11), and 125 (23.6%) had poor symptom control (score of 0–7).

When UCT scores were assessed according to a current or previous history of angioedema, patients without angioedema had a significantly higher mean ± SD UCT score (10.1 ± 3.4) than patients with a known history of angioedema (8.1 ± 3.3; *p* < 0.001; Table 1). Based on prescribed medications, patients receiving antihistamines (*n* = 349; 66.0%) at the time of completing the questionnaire had a numerically higher mean ± SD UCT score (9.8 ± 3.5) than those receiving other medications, including patients receiving topical corticosteroids (*n* = 185; 35.0%), who had a mean ± SD UCT score of 8.6 ± 3.2 (Table 2). Patients who were receiving omalizumab (*n* = 14; 2.6%) had a mean ± SD UCT score of 7.9 ± 3.5 (Table 2).

Among patients on current antihistamine therapy, patients receiving antihistamines alone had significantly higher UCT scores than those receiving antihistamines in combination with other drugs (mean ± SD 11.2 ± 3.2 vs. 9.2 ± 3.4; *p* < 0.001; Table 3). In addition, patients currently receiving an increased antihistamine dose had a significantly lower mean ± SD UCT score (7.8 ± 3.7) than those with a previous dose increase (9.7 ± 3.4; *p* < 0.001) or those with no dose escalation (10.6 ± 3.2; *p* < 0.001).

There was also a significant trend towards lower UCT scores in patients receiving a higher number of current medications (*p* < 0.001 for decreasing trend). For example, patients receiving six or more current medications (*n* = 44; 8.3%) had a mean ± SD UCT score of 7.1 ± 3.2, whereas those receiving one or no current medications had mean ± SD UCT scores of 10.6 ± 3.3 and 11.6 ± 3.6, respectively (Table 3).

### 3.3. Treatment Satisfaction

**Figure 3:**
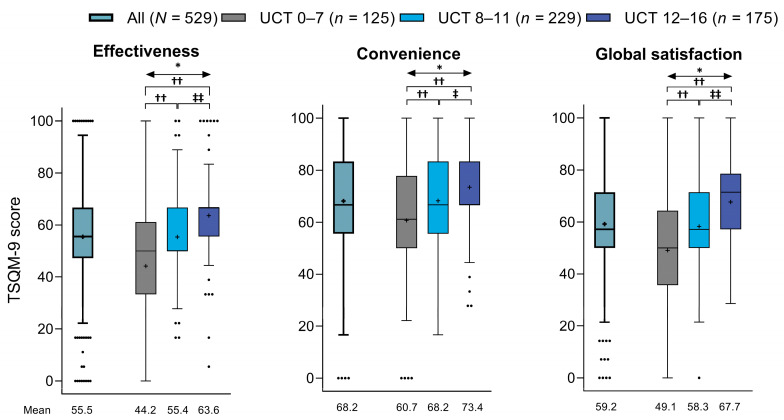
TSQM-9 scores for effectiveness, convenience, and global satisfaction in all patients and in subgroups based on UCT scores (Tukey box plot). For each plot, the box represents the lower quartile (Q1), median, and upper quartile (Q3) values, the cross represents the mean value, the error bars represent the minimum/maximum values (excluding outliers), and the dots represent the outliers. * *p* < 0.01 across UCT subgroups (Kruskal–Wallis test): †† *p* < 0.01 vs. UCT 0–7 subgroup (Steel–Dwass test); ‡ *p* < 0.05 vs. UCT 8–11 subgroup (Steel–Dwass test); and ‡‡ *p* < 0.01 vs. UCT 8–11 subgroup (Steel–Dwass test). TSQM-9 = Treatment Satisfaction Questionnaire for Medication-9 item; UCT = Urticaria Control Test.

Patients had mean ± SD TSQM-9 scores of 55.5 ± 17.6% for the effectiveness domain, 68.2 ± 18.8% for the convenience domain, and 59.2 ± 18.4% for the global satisfaction domain (Figure 3). According to these scores, treatment satisfaction was significantly lower in patients with poor (UCT score of 0–7) or insufficient (UCT score of 8–11) symptom control than in those with good symptom control (UCT score of 12–16) across all three TSQM-9 domains (convenience score *p* < 0.05 for UCT 8–11 vs. UCT 12–16 subgroup; *p* < 0.01 for all other comparisons).

When treatment satisfaction was assessed according to current medications, antihistamine use was associated with numerically higher mean ± SD TSQM-9 scores for effectiveness (56.0 ± 16.1%), convenience (69.7 ± 18.5%), and global satisfaction (59.6 ± 18.0%) than other medications (Table 2). Patients using topical corticosteroids reported slightly lower treatment satisfaction across all three TSQM-9 domains than those receiving antihistamines. Among patients receiving omalizumab, the mean ± SD TSQM-9 scores were 50.8 ± 10.0% for effectiveness, 51.6 ± 17.4% for convenience, and 54.6 ± 13.9% for global satisfaction (Table 2).

In patients currently receiving regular antihistamine therapy, TSQM-9 scores for all three domains were significantly higher in patients on antihistamine monotherapy than in those taking antihistamines in combination with other drugs (*p* < 0.01 for effectiveness and convenience and *p* < 0.001 for global satisfaction; Table 3). Use of a higher number of medications was associated with slightly lower treatment satisfaction scores, although the trend was not statistically significant for any of the TSQM-9 domains. Patients receiving six or more medications (*n* = 44; 8.3%) had mean ± SD TSQM-9 scores of 47.0 ± 16.8% for effectiveness, 57.2 ± 20.0% for convenience, and 50.3 ± 19.7% for global satisfaction, compared with 58.5 ± 18.1%, 71.0 ± 18.3%, and 61.8 ± 18.1%, respectively, among those receiving one medication, and 57.6 ± 22.5%, 69.7 ± 20.0%, and 63.2 ± 21.2%, respectively, among those receiving no medications (Table 3).

### 3.4. Disease Burden

**Figure 4:**
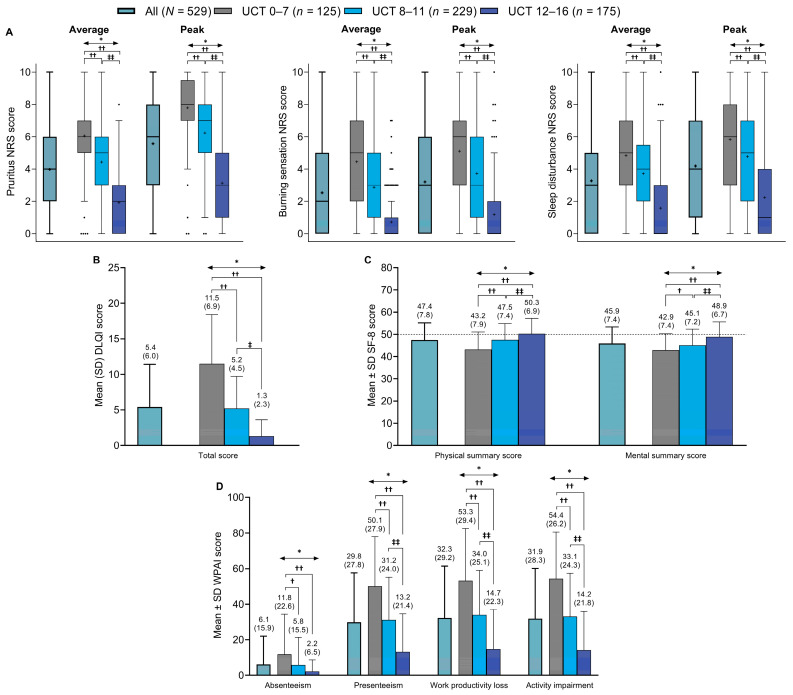
Summary of the scores in all patients and in subgroups based on UCT scores for: (**A**) average and maximum NRS scores for pruritus, burning sensation, and sleep disturbance (Tukey box plots, the dots represent the outliers), (**B**) DLQI total score, (**C**) SF-8 health survey physical and mental summary scores, and (**D**) WPAI scores for absenteeism, presenteeism, work productivity loss, and activity impairment domains. The dashed line in (**C**) represents the Japanese national standard mean SF-8 score (i.e., 50). * *p* < 0.01 across UCT subgroups (Kruskal–Wallis test): † *p* < 0.05 vs. UCT 0–7 subgroup (Steel–Dwass test); †† *p* < 0.01 vs. UCT 0–7 subgroup (Steel–Dwass test); ‡ *p* < 0.05 vs. UCT 8–11 subgroup (Steel–Dwass test); and ‡‡ *p* < 0.01 vs. UCT 8–11 subgroup (Steel–Dwass test). DLQI = Dermatology Life Quality Index; NRS = numerical rating scale; SD = standard deviation; SF-8 = Short Form-8; UCT = Urticaria Control Test; WPAI = Work Productivity and Activity Impairment.

Average and peak NRS scores for pruritus, burning sensation, and sleep disturbance across 7 days are shown in Figure 4A. All NRS scores were significantly higher (i.e., worse) in patients with poor (UCT score of 0–7) or insufficient (UCT score of 8–11) symptom control compared with good control (*p* < 0.01 for all comparisons). In patients with good symptom control (UCT score of 12–16), symptoms of burning sensations appeared to decrease or disappear, whereas pruritus and sleep disturbance persisted.

Regarding HRQoL, DLQI scores were significantly higher (i.e., HRQoL was poorer) in patients with poor or insufficient symptom control than in those with good symptom control (Figure 4B). Mean ± SD DLQI total scores were 11.5 ± 6.9 in patients with poor control (i.e., UCT score of 0–7), 5.2 ± 4.5 in patients with insufficient control (i.e., UCT score of 8–11; *p* < 0.01 vs. UCT 0–7 subgroup), and 1.3 ± 2.3 in patients with good control (i.e., UCT score of 12–16; *p* < 0.01 vs. UCT 0–7 subgroup and *p* < 0.01 vs. UCT 8–11 subgroup). Individual DLQI subscores were also significantly higher in the UCT 0–7 and UCT 8–11 subgroups than in the UCT 12–16 subgroup (Supplementary Figure S1).

Similarly, SF-8 physical and mental summary scores were significantly lower (i.e., indicating poorer HRQoL) in patients with poor or insufficient symptom control than in those with good symptom control, and lower than the mean ± SD national standard SF-8 scores for the Japanese general population (50 ± 10; Figure 4C). Mean ± SD physical and mental summary scores were 43.2 ± 7.9 and 42.9 ± 7.4, respectively, in patients with poor control (UCT score of 0–7), 47.5 ± 7.4 and 45.1 ± 7.2 in those with insufficient control (UCT score of 8–11; *p* < 0.01 and *p* < 0.05 vs. UCT 0–7 subgroup, respectively), and 50.3 ± 6.9 and 48.9 ± 6.7 in those with good control (UCT score of 12–16; both *p* < 0.01 vs. UCT 0–7 subgroup and UCT 8–11 subgroup, respectively).

Mean ± SD WPAI scores across a period of 7 days were low for absenteeism (i.e., fewer lost work hours) in all patients and across UCT subgroups, ranging from 2.2 ± 6.5% in patients with good symptom control (UCT score of 12–16) to 11.8 ± 22.6% in those with poor symptom control (UCT score of 0–7; *p* < 0.01; Figure 4D). WPAI absenteeism scores were also significantly lower in patients with insufficient control (UCT score of 8–11) than in those with poor control (*p* < 0.05). For the other three WPAI items (presenteeism, lost work productivity, and activity impairment), mean ± SD scores ranged from 50.1 ± 27.9% to 54.4 ± 26.2% in patients with poor symptom control, 31.2 ± 24.0% to 34.0 ± 25.1% in those with insufficient control, and 13.2 ± 21.4% to 14.7 ± 22.3% in those with good control. Scores for these three WPAI items were significantly lower in patients with good control than in those with poor control or insufficient control (*p* < 0.01 for both comparisons), and were significantly lower in patients with insufficient control than in those with poor control (*p* < 0.01).

## 4. Discussion

### 4.1. Overview

In this web-based observational study of Japanese patients with CSU, many patients had longstanding disease (mean ± SD disease duration of 13.2 ± 13.0 years), and two-thirds had insufficient or poor symptom control based on UCT scores, a validated, easy-to-use tool that determines disease control in patients with all subforms of CU [11]. Treatment satisfaction tended to be lower among those with poorer symptom control (i.e., lower UCT scores) overall and across TSQM-9 effectiveness, convenience, and global satisfaction domains. Patients with poor or insufficient symptom control also reported an increased disease burden, numerically higher pruritus, burning sensation, and sleep disturbance NRS scores, higher DLQI scores, lower SF-8 scores, and higher WPAI scores compared to those with good symptom control.

### 4.2. Epidemiology

In this study, confirmed diagnoses of CSU and CU were reported by 16.4% and 93.8% of patients, respectively. However, it should be noted that the disease term ‘CU’ in Japan is regarded as being almost identical to the international ‘CSU’ disease term (i.e., Japanese guidelines do not use the term ‘CU’) because the guidelines classify acute urticaria and CU after diagnosing spontaneous urticaria [1]. As such, it is important to note that in this study, we considered a diagnosis of CU as CSU if patients had indicated typical characteristics of CSU (i.e., experienced urticaria symptoms for >6 weeks with no previous identifiable triggers). In addition, 42.5% of patients in this study reported a diagnosis of allergic urticaria. The study by Saito and colleagues reported that 66.8% of 1061 patients with urticaria were classified as having CSU in 2020, and only 0.8% as having allergic urticaria (i.e., mediated by type I hypersensitivity) [5], the latter of which was consistent with the allergic urticaria prevalence reported by a 2020 national patient survey from the Ministry of Health, Labor, and Welfare [15]. This indicates that, in the current study, over 40% of patients and/or their physicians incorrectly identified the cause of their CSU or CU as being allergic in nature (i.e., allergen/IgE-mediated), despite having symptoms consistent with the definition of CSU (i.e., having no identifiable trigger). Together, these findings highlight the need to further educate both physicians and patients to raise disease awareness, facilitate easier CSU diagnosis, and increase the recognition of the international CSU disease term in Japan. In the general population of Japanese residents aged ≥20 years who responded to this online survey, the estimated point prevalence of CSU was 0.4%, which is similar to the previously reported urticaria point prevalence of 0.8% in China [3], with approximately two-thirds of patients with urticaria having CSU, as reported in the previous Japanese study by Saito and colleagues [5]. In this study population, patients with CSU had a mean age of 45.3 years, and 59.9% were female, which is similar to the demographics of previous real-world studies from Japan [5,6] and Western countries [16].

The proportion of patients with a previous or current history of angioedema in our current study (12.1%) was lower than that previously reported in the ASSURE-CSU study in Western populations (40.3%) [17]. In contrast, the randomized, double-blind, placebo-controlled phase III POLARIS trial of omalizumab treatment in an East Asian population with CSU reported angioedema in 16.4%–20.3% of its treatment groups [18]. Similarly, the 2020 Japanese epidemiology study reported angioedema in 14.1% of patients with urticaria [5]. Therefore, our finding regarding the low prevalence of angioedema is in line with previous publications, indicating that angioedema is less common in Asian versus Western populations with CSU. In the present study, patients with angioedema had significantly worse symptom control than those with no history of angioedema. This is in line with results from ASSURE-CSU, where disease severity and activity increased as the incidence of angioedema increased [17].

### 4.3. Treatment Satisfaction

Since the 2018 study by Itakura and colleagues [6], there have been no reports of a relationship between urticaria control status and treatment satisfaction; however, the treatment of urticaria in Japan has advanced in more recent years. Treatment satisfaction for all patients in the current study (mean TSQM-9 scores of 55.5% for effectiveness, 68.2% for convenience, and 59.2% for global satisfaction) was notably lower than reported in the previous survey of 90 Japanese patients with CSU by Kaneko and colleagues (TSQM-9 scores of 68.6%, 72.0%, and 72.2%, respectively) [9]. This discrepancy may be due to differences in therapeutic and explanatory approaches, as patients in the previous study were seen at specialist dermatology departments [9], while those in the current study were treated in various dermatology and non-dermatology clinics and hospital departments.

In the current study, patients who were prescribed oral antihistamines had higher treatment satisfaction than those receiving other medications. Overall, inadequate symptom control is generally linked to lower treatment satisfaction in patients with CU [19]. Patients who were only prescribed oral antihistamines may have milder and better controlled symptoms. Although the Japanese urticaria management guidelines recommend second-generation antihistamines (H1 receptor antagonists) as a first-line treatment [1], 13.2% of patients in our study reported never receiving prescribed oral antihistamines, potentially because they had only taken over-the-counter medications without consulting a physician or had only been prescribed topical medications.

Despite the high frequency of prescribed topical corticosteroid use in the current study (35.0% of patients), this treatment was associated with lower UCT scores and treatment satisfaction than antihistamines. According to the Japanese and international guidelines for urticaria management, recommended treatment options do not include topical corticosteroids [1,2]; this is due to a lack of evidence of their efficacy in treating CSU, which may explain the lower UCT scores and low treatment satisfaction among patients using topical corticosteroids in our study. In contrast to our results, a previous Japanese survey of 90 patients with CSU seen at specialist dermatology departments showed that the prescription of topical corticosteroids is broadly in line with guideline recommendations, with only 3/90 patients (3%) using topical corticosteroids [9]. While it is not clear why there is such a difference in the prescribing rate of topical steroids (35% vs. 3%), our results indicate a need to increase awareness among physicians of the standard guideline-directed therapies, as well as a need for more efficacious treatment options, which should lead to improved treatment satisfaction.

In the current study, patients had numerically lower treatment satisfaction scores than those reported previously by Kaneko and colleagues [9], and patients receiving omalizumab had similar treatment satisfaction to those receiving other drugs. The lower treatment satisfaction observed in our study may be because the Kaneko et al. study was conducted in specialist dermatology departments, whereas our study evaluated treatment satisfaction in a real-world setting that included patients who did not receive expert medical care. However, particularly as the number of patients receiving omalizumab in the current study was low (*n* = 14; 2.6%), these findings and comparisons between other studies should be interpreted with caution.

### 4.4. Disease Burden

Patients with CSU are known to have a high disease burden [6,9]. In the current study, pruritus average NRS scores were numerically lower than peak NRS scores. This may be a characteristic of urticaria, in which the transient erythema, wheals, and pruritus symptoms often appear and fade repeatedly [5]. Pruritus, burning sensation, and sleep disturbance NRS scores were significantly higher among patients with lower UCT scores (i.e., poor symptom control). Of note, burning sensation is not typically assessed in patients with CSU, although this symptom may become more widely evaluated and reported in patients with CSU.

As expected, DLQI and SF-8 scores indicated that patients with poor (i.e., UCT score of 0–7) or insufficient (i.e., UCT score of 8–11) symptom control had significantly lower HRQoL than those with good control (i.e., UCT score of 12–16). Similarly, a previous study of Japanese patients with CSU reported a strong inverse correlation between UCT and DLQI scores (*r_s_* = –0.8349; *p* < 0.0001, *n* = 80), indicating that UCT is a reliable measure to assess HRQoL in adult Japanese patients with CSU [20].

In the current study, patients had low overall absenteeism scores, indicating that few patients missed work due to urticaria symptoms. In a previous web survey of Japanese patients with CU (*n* = 409), the mean ± SD absenteeism score was 2.4 ± 9.0 overall, 2.9 ± 9.0 in the UCT 0–7 subgroup, 1.7 ± 6.9 in the UCT 8–11 subgroup, and 2.8 ± 10.7 in the UCT 12–16 subgroup, with no trend observed according to the UCT score [6]. In contrast, the current study showed a higher overall absenteeism score (6.1 ± 15.9) than that reported in the previous web survey [6], and found significantly higher absenteeism scores in patients with poor (UCT score of 0–7) versus insufficient (score of 8–11) or good (score of 12–16) symptom control. One possible explanation for this divergence between study results may be a difference in study populations. While the previous survey included patients with CU (defined as the presence of chronic symptoms (wheals, itching, and angioedema) persisting for >6 weeks) [6], the current study evaluated patients with CSU (defined as physician-diagnosed CSU or CU, with urticaria symptoms for >6 weeks with no previous trigger). Another reason may be a change in the CSU disease terminology over time. As the disease term ‘CSU’ has become more widely used and patients have become more accurately diagnosed in the past few years, patients with CSU (i.e., CU with no previous trigger) may have become more aware of their condition.

### 4.5. Limitations

The limitations of this study included those inherent to web-based questionnaires, such as that all study outcomes were PROs, and the possible influence of selection bias (participants could choose whether they responded to the questionnaire) and recall bias. However, while these limitations exist, this method of investigation has been employed in several studies previously [19,21,22], as studies of this type can provide insight into the real-world management and treatment of a disease. Additionally, given the small number of participants receiving omalizumab in the current study, findings regarding treatment satisfaction with this medication should be interpreted with caution.

## 5. Conclusions

This web-based questionnaire of patients with CSU in Japan found that disease burden was high, whereas treatment satisfaction was not high and showed no major differences when assessed according to current medications. To improve treatment satisfaction and reduce disease burden in patients with CSU, improved disease control is needed, as higher UCT scores correlate with higher treatment satisfaction and lower disease burden. Good disease control can be achieved with accurate diagnosis and appropriate therapy; thus, physicians should follow guideline-based treatment strategies. The current study found that topical steroids are frequently used, despite the lack of recommendations from either the Japanese or international guidelines, indicating that standardized treatment, as well as better treatment options, are needed for patients with CSU in Japan.

## Figures and Tables

**Table 1:** Patient demographics, baseline characteristics, and UCT scores for each category.

Item	Study Cohort *n* = 529	UCT Score (Mean ± SD)
Total population, *n* (%)	529 (100.0)	9.7 ± 3.4
Sex, *n* (%)		
Male	212 (40.1)	9.5 ± 3.4
Female	317 (59.9)	9.9 ± 3.5
Age, years (mean ± SD)	45.3 ± 13.2	
Age group, years, *n* (%)		
20–29	76 (14.4)	8.7 ± 3.4
30–39	107 (20.2)	9.0 ± 3.3
40–49	152 (28.7)	9.5 ± 3.5
50–59	104 (19.7)	10.5 ± 3.5
≥60	90 (17.0)	10.9 ± 3.0
Age at urticaria diagnosis, years (mean ± SD)	32.1 ± 15.6	
Age group at urticaria diagnosis, years, *n* (%)		
0–19	106 (20.0)	8.5 ± 3.5
20–29	135 (25.5)	9.2 ± 3.6
30–39	120 (22.7)	10.1 ± 3.2
40–49	81 (15.3)	10.4 ± 3.2
50–59	61 (11.5)	10.8 ± 3.5
≥60	26 (4.9)	11.2 ± 2.8
Specialty of diagnosing physician, *n* (%)		
Dermatologist	441 (83.4)	9.8 ± 3.4
Internal medicine physician	58 (11.0)	9.9 ± 3.6
Other	25 (4.7)	8.4 ± 3.6
Unknown	5 (0.9)	9.2 ± 3.4
Duration of disease, years (mean ± SD)	13.2 ± 13.0	
Duration of disease, years, *n* (%)		
<1	77 (14.6)	9.1 ± 3.6
≥1 to <5	150 (28.4)	10.0 ± 3.4
≥5 to <10	96 (18.1)	9.6 ± 3.6
≥10 to <15	67 (12.7)	10.4 ± 3.1
≥15 to <20	36 (6.8)	9.8 ± 3.2
≥20 to <30	56 (10.6)	9.5 ± 3.4
≥30	47 (8.9)	9.6 ± 3.5
Types of CU diagnosed, ^1^ *n* (%)		
CU	496 (93.8)	
CSU	87 (16.4)	
Other types of urticaria diagnosed, *n* (%)		
Allergic urticaria ^2^	225 (42.5)	
Acute urticaria	164 (31.0)	
Cold urticaria	95 (18.0)	
Other	44 (8.3)	
Current or previous history of angioedema, *n* (%)		
Yes	64 (12.1)	8.1 ± 3.3 *
No	407 (76.9)	10.1 ± 3.4
Unknown	58 (11.0)	9.3 ± 3.7

^1^ Patients could choose more than one response. ^2^ An allergen/IgE-mediated urticaria subtype that occurs due to exposure to food, drugs, plants (including natural rubber products), insect toxins, etc., as defined by the Japanese guidelines [1]. * *p* < 0.001 vs. no angioedema (Mann–Whitney U test); test performed ad hoc for the presence vs. absence of angioedema only in this table. CU = chronic urticaria; CSU = chronic spontaneous urticaria; IgE = immunoglobulin E; SD = standard deviation; UCT = Urticaria Control Tool.

**Table 2:** UCT and TSQM-9 scores according to patient current medication use (N = 529).

	*n* (%)	UCT Score (Mean ± SD)	TSQM-9 Score (Mean ± SD)		
			Effectiveness	Convenience	Global Satisfaction
Total population	529 (100.0)	9.7 ± 3.4	55.5 ± 17.6	68.2 ± 18.8	59.2 ± 18.4
OTC medications ^1^					
Oral	130 (24.6)	9.4 ± 3.2	55.1 ± 16.4	68.5 ± 18.0	58.7 ± 18.9
Topical	107 (20.2)	8.1 ± 3.1	50.1 ± 17.4	63.2 ± 18.0	56.2 ± 17.9
Prescribed medications ^1^					
Oral medications					
Antihistamines	349 (66.0) ^2^	9.8 ± 3.5	56.0 ± 16.1	69.7 ± 18.5	59.6 ± 18.0
OCS	64 (12.1)	7.8 ± 3.6	50.3 ± 17.8	63.7 ± 19.9	55.2 ± 20.9
Chinese herbal medicines	62 (11.7)	8.2 ± 3.5	49.0 ± 18.7	62.5 ± 20.2	55.4 ± 20.4
Hypnotics/sleep inducers	52 (9.8)	8.2 ± 3.6	48.3 ± 17.1	64.1 ± 20.4	50.4 ± 18.5
Oral tranexamic acid	45 (8.5)	8.2 ± 3.4	51.9 ± 20.4	64.3 ± 21.1	55.7 ± 19.1
Antidepressants/anxiolytics	44 (8.3)	7.4 ± 2.8	49.4 ± 14.0	62.9 ± 20.4	51.3 ± 17.0
Oral H2 receptor antagonists	31 (5.9)	8.9 ± 3.7	50.2 ± 16.4	62.2 ± 22.4	55.5 ± 14.7
Oral leukotriene receptor antagonists	28 (5.3)	8.2 ± 3.8	50.8 ± 13.3	62.9 ± 18.5	54.6 ± 13.2
Cyclosporine	17 (3.2)	7.8 ± 3.3	50.0 ± 14.8	59.2 ± 21.9	50.8 ± 14.3
Diaphenylsulfone	15 (2.8)	7.4 ± 3.6	48.1 ± 19.1	66.7 ± 20.0	53.8 ± 17.9
Topical drugs					
TCS	185 (35.0)	8.6 ± 3.2	53.2 ± 17.8	65.1 ± 18.4	56.4 ± 18.5
Tacrolimus ointment	56 (10.6)	8.5 ± 3.2	50.0 ± 17.1	56.5 ± 17.3	53.6 ± 16.6
Injectables					
Tranexamic acid	14 (2.6)	8.1 ± 4.2	50.8 ± 8.4	52.8 ± 16.1	54.1 ± 12.4
Omalizumab	14 (2.6)	7.9 ± 3.5	50.8 ± 10.0	51.6 ± 17.4	54.6 ± 13.9
Glycyrrhizin/glycine/cysteine	13 (2.5)	6.8 ± 2.9	46.2 ± 18.6	49.6 ± 22.4	47.8 ± 20.5
H2 receptor antagonists	11 (2.1)	5.6 ± 2.9	43.9 ± 21.1	40.9 ± 20.7	46.8 ± 27.4
Neutropin ^3^	11 (2.1)	6.9 ± 2.7	46.0 ± 18.1	42.4 ± 19.8	46.8 ± 19.3
Other	20 (3.8)	9.3 ± 4.1	52.2 ± 24.0	65.3 ± 23.1	58.6 ± 27.1

^1^ Patients could choose more than one response; ^2^ The remaining 34.0% of patients not using prescription antihistamines were either untreated or using ≥2 of the drugs listed above. As all OTC medications were classified as either oral or topical OTCs, some patients may have been using OTC antihistamines; ^3^ Extract of inflamed skin from vaccinia virus-inoculated rabbits. OCS = oral corticosteroids; OTC = over-the-counter; SD = standard deviation; TCS = topical corticosteroids; TSQM-9 = Treatment Satisfaction Questionnaire for Medication–9 items; UCT = Urticaria Control Tool.

**Table 3:** UCT and TSQM-9 scores according to patient treatment status, antihistamine dose status, healthcare institute, and number of medications (*N* = 529).

	*n* (%)	UCT Score (Mean ± SD)	TSQM-9 Scores (Mean ± SD)		
			Effectiveness	Convenience	Global Satisfaction
Antihistamine treatment status					
Current regular antihistamine therapy ^1^	349 (66.0)	9.8 ± 3.5	56.0 ± 16.1	69.7 ± 18.5	59.6 ± 18.0
As monotherapy	117 (22.1)	11.2 ± 3.2 **	59.9 ± 15.7 *	74.3 ± 16.8 *	64.6 ± 16.8 **
In combination with other drugs	232 (43.9)	9.2 ± 3.4	54.0 ± 16.0	67.4 ± 18.9	57.1 ± 18.1
Previously treated	110 (20.8)	9.7 ± 3.3	54.4 ± 21.2	63.9 ± 18.7	58.2 ± 20.1
Never received	70 (13.2)	9.2 ± 3.5	54.3 ± 18.8	67.1 ± 19.5	58.8 ± 17.9
Antihistamine dose status ^2^					
Dose currently being increased	67 (14.6)	7.8 ± 3.7	50.1 ± 20.2	64.0 ± 23.5	55.4 ± 20.1
Dose previously increased	149 (32.5)	9.7 ± 3.4 ^†^	54.1 ± 16.4	66.3 ± 18.1	57.7 ± 17.8
No dose increase	200 (43.6)	10.6 ± 3.2 ^†^	59.1 ± 16.8 ^†,‡^	71.6 ± 17.7 ^‡,§^	63.1 ± 18.6 ^‡,§^
Unknown	43 (9.4)	9.9 ± 3.4	53.6 ± 16.9	67.1 ± 14.1	53.3 ± 14.2
TCS treatment status					
Current regular treatment ^1^	185 (35.0)	8.6 ± 3.2	53.2 ± 17.8	65.1 ± 18.4	56.4 ± 18.5
Previous treatment	169 (31.9)	10.1 ± 3.4	53.7 ± 17.1	66.5 ± 19.1	58.0 ± 17.1
Never received	175 (33.1)	10.7 ± 3.4	59.4 ± 17.3	73.0 ± 18.1	63.3 ± 19.0
Healthcare institute					
Dermatology clinic	358 (67.7)	9.6 ± 3.3	55.7 ± 17.1	67.7 ± 18.7	59.4 ± 18.5
Hospital, dermatology department	55 (10.4)	8.9 ± 3.6	52.5 ± 17.0	64.6 ± 20.2	58.2 ± 16.2
Non-dermatology clinic	62 (11.7)	10.4 ± 3.5	57.0 ± 16.9	71.9 ± 19.2	61.8 ± 20.0
Hospital, non-dermatology department	11 (2.1)	8.1 ± 3.0	41.9 ± 18.8	53.0 ± 14.6	50.6 ± 12.1
Other	10 (1.9)	10.8 ± 3.0	64.4 ± 14.9	75.6 ± 14.6	67.9 ± 14.4
No. of current medications					
0	40 (7.6)	11.6 ± 3.6	57.6 ± 22.5	69.7 ± 20.0	63.2 ± 21.2
1	186 (35.2)	10.6 ± 3.3	58.5 ± 18.1	71.0 ± 18.3	61.8 ± 18.1
2	134 (25.3)	9.5 ± 3.2	55.1 ± 15.6	67.2 ± 19.1	56.4 ± 18.0
3	69 (13.0)	9.2 ± 3.3	52.1 ± 17.2	70.6 ± 17.4	60.1 ± 17.0
4	38 (7.2)	9.2 ± 3.5	57.5 ± 14.9	67.3 ± 15.3	61.3 ± 14.8
5	18 (3.4)	8.2 ± 2.3	51.5 ± 16.3	62.7 ± 18.2	58.7 ± 20.0
≥6	44 (8.3)	7.1 ± 3.2 ^#^	47.0 ± 16.8	57.2 ± 20.0	50.3 ± 19.7

^1^ As presented in Table 2. ^2^ Denominator of *n* = 459 used for percent calculations. * *p* < 0.01 vs. antihistamines in combination with other drugs (Mann–Whitney U test). ** *p* < 0.001 vs. antihistamines in combination with other drugs (Mann–Whitney U test). ^†^ *p* < 0.001 vs. current antihistamine dose increase (Steel–Dwass test). ^‡^ *p* < 0.05 vs. previous antihistamine dose increase (Steel-Dwass U test). ^§^ *p* < 0.05 vs. current antihistamine dose increase (Steel-Dwass U test). ^#^ *p* < 0.001 for decreasing trend (Jonckheere–Terpstra test). No. = number; SD = standard deviation; TCS = topical corticosteroids; TSQM-9 = Treatment Satisfaction Questionnaire for Medication–9 items; UCT = Urticaria Control Tool.

## Data Availability

The data that support the findings of this study are available from the corresponding author upon reasonable request.

## Supplementary Materials

The following supporting information can be downloaded at: https://www.mdpi.com/article/10.3390/jcm13102967/s1, Table S1: Currently treated comorbidities in the study cohort; Figure S1: Summary of scores in all patients and in subgroups based on UCT for DLQI sub-item scores.
