# Supplementary material for: Disease Control and Treatment Satisfaction in Patients with Chronic Spontaneous Urticaria in Japan
Source: J Clin Med. 2024 May 17;13(10):2967. doi: 10.3390/jcm13102967 (PMC11121873; doi:10.3390/jcm13102967)
Supplement: Supplementary file 1 [file jcm-13-02967-s001.zip › jcm-2957972-supplementary.pdf]

Disease control and treatment satisfaction in patients with chronic spontaneous urticaria in Japan

Table S1. Currently treated comorbidities in the study cohort

**Item,[1] *n* (%)**	**Study cohort (*N* = 529)**
**Allergic conditions**	
Allergic rhinitis	143 (27.0)
Atopic dermatitis	95 (18.0)
Allergic contact dermatitis	58 (11.0)
Allergic conjunctivitis	47 (8.9)
Bronchial asthma	43 (8.1)
Food allergy	42 (7.9)
Chronic sinusitis	40 (7.6)
Drug allergy	13 (2.5)
Eosinophilic esophagitis	4 (0.8)
Eosinophilic gastroenteritis	3 (0.6)
**Lifestyle-related diseases**	
Hypertension	84 (15.9)
Hyperlipidemia	50 (9.5)
Diabetes	24 (4.5)
Osteoporosis	12 (2.3)
**Psychological disorders**	
Insomnia	57 (10.8)
Depression	44 (8.3)
Anxiety	32 (6.0)
**Autoimmune disorders**	
Thyroid disease	23 (4.3)

Alopecia areata	15 (2.8)
Rheumatoid arthritis	15 (2.8)
Ulcerative colitis	12 (2.3)
Other collagen diseases	9 (1.7)
Crohn's disease	3 (0.6)
Not being treated for any of the above	209 (39.5)

1—Patients could choose more than one response.

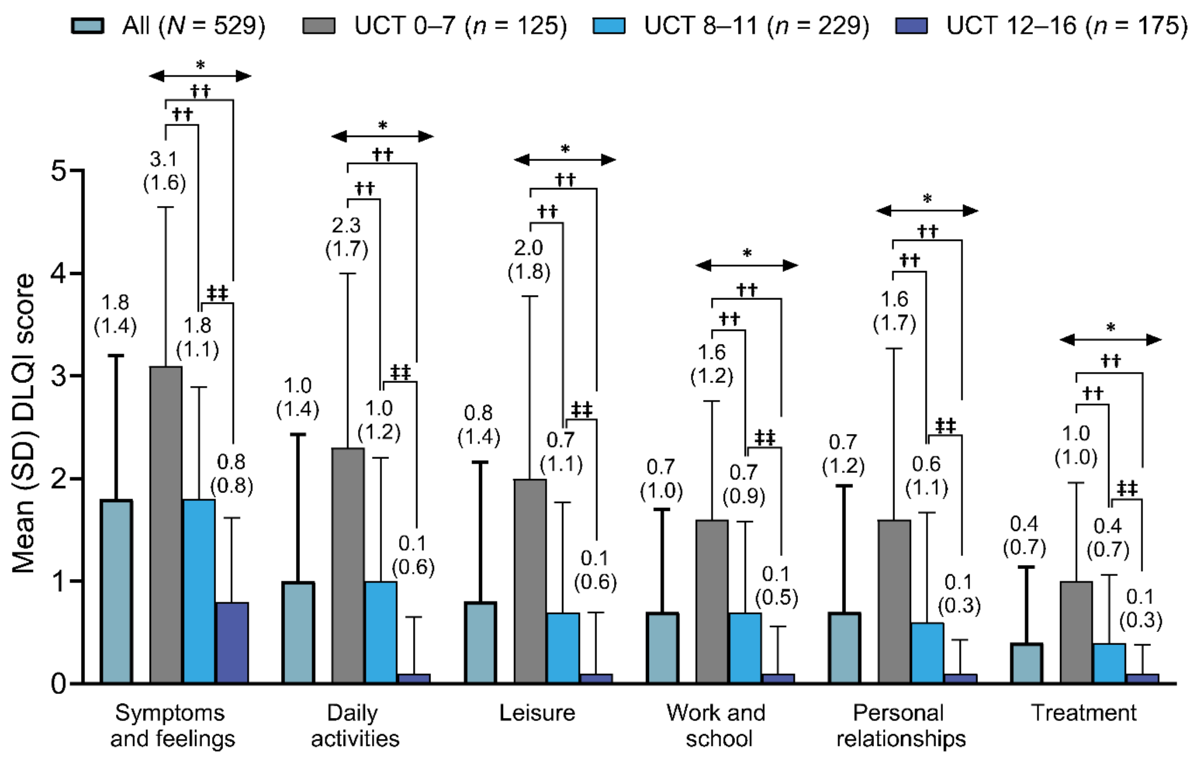

Figure S1. Summary of scores in all patients and in subgroups based on UCT for DLQI sub-item scores. *p < 0.01 across UCT subgroups (Kruskal–Wallis test); ††p < 0.01 vs. UCT 0–7 subgroup (Steel–Dwass test); ‡‡p < 0.01 vs. UCT 8–11 subgroup (Steel–Dwass test). Abbreviations: DLQI—Dermatology Life Quality Index; SD—standard deviation; UCT—Urticaria Control Test
